# Incidence and treatment strategy for patellar instability in Norway

**DOI:** 10.1002/jeo2.70519

**Published:** 2025-11-14

**Authors:** Trine Hysing‐Dahl, Håvard Visnes, Truls M. Straume‐Næsheim, Per A. S. Waaler, Asle Kjellsen, Henning A. Warø, Christian Øye, Ann K. Hansen, Alexander Tandberg, Andreas Persson, Eivind Inderhaug

**Affiliations:** ^1^ Department of Clinical Medicine University of Bergen Bergen Norway; ^2^ Department of Orthopaedic Surgery Haraldsplass Deaconal Hospital Bergen Norway; ^3^ Faculty of Health and Social Sciences Western Norway University of Applied Sciences Bergen Norway; ^4^ Department of Sport and Outdoor Studies Oslo Sports Trauma Research Center, Norwegian School of Sports Science Oslo Norway; ^5^ The Norwegian Knee Ligament Register, Department of Orthopedic Surgery Haukeland University Hospital Bergen Norway; ^6^ Department of Orthopedic Surgery Sørlandet Hospital Kristiansand Norway; ^7^ Department of Orthopaedic Surgery Akershus University Hospital Oslo Norway; ^8^ Department of Orthopaedic Surgery Haukeland University Hospital Bergen Norway; ^9^ Department of Orthopaedic Surgery St. Olavs University Hospital Trondheim Norway; ^10^ Department of Orthopaedic Surgery University Hospital of North Norway Tromsø Norway; ^11^ Department of Clinical Medicine The Arctic University of Norway Tromsø Norway; ^12^ Department of Orthopaedic Surgery Martina Hansens Hospital Oslo Norway; ^13^ Department of Orthopedic Surgery Aker Oslo University Hospital Oslo Norway

**Keywords:** medial patellofemoral ligament, patellar instability, rehabilitation, trochleoplasty, tuberosity tibia osteotomy

## Abstract

**Purpose:**

To determine the incidence and treatment strategy for patellar instability in the Norwegian healthcare system.

**Methods:**

Clinicians from all regional health regions were invited to contribute to the development of a cross‐sectional survey of the current national clinical practices for managing this patient group. A tailored questionnaire was developed to determine how many hospitals in Norway performed patellar stabilization surgery and which surgical procedures and postoperative routines were used. Specifically, the following variables were included: number of procedures performed in 2023, types of procedures, treatment of children, choice of graft for primary MPFL reconstruction, routine use of orthoses and postoperative restrictions.

**Results:**

A survey was conducted among 68 hospitals, of which 35 reported performing patellar stabilisation procedures. The total number of procedures recorded was 532, yielding an incidence rate of 9.6 per 100,000 people. The number of procedures varied significantly among hospitals, with most performing between five and 20. All hospitals conducted medial patellofemoral ligament reconstruction, while 28 included bony procedures, and only seven performed derotational osteotomies. The preferred graft was gracilis (89%), and the most common fixation method on the femur was intrerference screw (60%). Postoperatively, only 20% of hospitals routinely recommended orthoses, primarily after tibial tuberosity osteotomy (86%). Restrictions on knee flexion were advised by 86% of respondents for up to eight weeks, and weight‐bearing restrictions were recommended by all after tibial tuberosity osteotomy.

**Conclusions:**

This study offers an overview of patellar instability incidence and treatment in Norway, revealing significant variability in surgeries, procedures, restrictions, and follow‐up practices. This underscores the need for standardized guidelines and a national registry to improve patient outcomes and care quality. Future research should focus on evaluating long‐term outcomes of various surgical approaches and protocols to inform best practices.

**Level of Evidence:**

Level III.

**Abbreviations**
MPFL: medial patellofemoral ligament
NKLR: Norwegian Knee Ligament Register
SD: standard deviations
TTO: tibial tubercle osteotomy

## INTRODUCTION

Recurrent patellar dislocation is a common disorder primarily affecting adolescents and young adults, with an incidence rate of 21.6 and 49.7 per 100,000 people [10, 24, 26, 27]. Following the initial dislocation, 23%–40% of patients experience recurrent dislocations, placing them at high risk of developing chronic patellar instability (PI) [8, 10, 17]. The great variation in injury mechanisms, rate of recurrent instability, underlying risk factors and levels of activity makes this patient group highly heterogeneous [2, 28, 33].

The consequences of patellar instability are extensive, influencing various aspects of life such as functional limitations and pain [7, 20, 31], wellbeing and overall quality of life in these young patients [3, 9, 14, 31]. Recent studies have demonstrated the profound negative impact of the disease on patients life [14, 20], yet they wait five times longer for surgical treatment compared to patients with similar knee disorders such as anterior cruciate ligament tears [31].

The complex aetiology of patellar instability makes it a challenging disorder to manage. The current standard of care following a first‐time dislocation in patients without osteochondral fractures or loose fragments is nonoperative management with exercise therapy [4, 6, 18, 29, 30]. Patellar stabilizing surgery is recommended for individuals experiencing recurrent dislocations regardless of their functional activity level [1, 6, 18, 29]. Despite the recognition that surgical interventions are necessary for many patients with recurrent patellar dislocation, there is an ongoing debate about when and how to treat these patients. Most algorithms, however, include reconstruction of the medial patellofemoral ligament (MPFL) either as an isolated procedure, or in combination with additional procedures such as tibial tubercle osteotomy (TTO), trochleoplasty and/or derotational osteotomies. However, there is no consensus about the necessity and indication of the additional procedures. This lack of consensus on surgical approaches leads to substantial variation in treatment. The diversity of surgical techniques and substantial variability in treatment strategies add further complexity to the decision‐making process regarding the most appropriate therapeutic approach. Therefore, the best way to treat this patient group is unclear [6, 18, 29, 30].

Currently, there is no comprehensive overview of treatment strategies across the Norwegian healthcare system, and the extent of unwarranted treatment variation is unclear. There is also a lack of knowledge regarding the epidemiology of patellar instability and the long‐term effects of living with such a disorder. A reasonable strategy to address these issues is to establish a national registry for patients with patellar instability. The purpose of such a national registry is to provide reliable and representative data for epidemiological and observational studies, thereby improving treatment options and complementing the existing literature in order to provide patients with high‐quality evidence‐based care. The Norwegian Knee Ligament Register (NKLR), established in 2004, was the first ligament register in the world. Since then, the NKLR has changed clinical practice in many areas in Norway based on research findings [23, 25]. The current study is the first step in developing a national registry for patients with patellar instability, aiming to determine the incidence and treatment strategy for patellar instability in the Norwegian healthcare system.

## MATERIALS AND METHODS

At the initiative of the NKLR, researchers and clinicians from all regional health regions were invited to contribute to the development of a cross‐sectional survey of the current national clinical practices for managing this patient group. A tailored questionnaire (Appendix 1) was developed to determine the number of hospitals in Norway performing patella stabilizing surgery and which surgical procedures and postoperative routines that were used. Specifically, the following variables were included: the number of procedures performed in 2023, types of procedures, treatment of children, choice of graft for primary MPFL reconstruction, use of orthosis and postoperative restrictions (Appendix 1).

**Figure 1:**
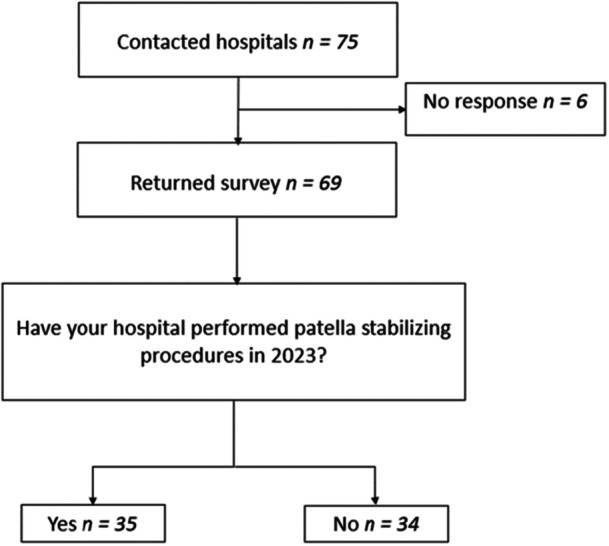
Flow chart of included hospitals.

Contact information for 75 hospitals was obtained from the NKLR, and an online survey was sent to all Norwegian hospitals with orthopaedic departments (Figure 1). All hospitals were contacted by email, and if no response was received, one of the authors contacted them by telephone.

### Statistics

SPSS 29.0 (IBM Corp.) was used for data analyses. These included descriptive statistics; for continuous variables, means and standard deviations (SD) are presented and for categorical variables absolute and relative frequencies are presented.

### Ethics

No patients were involved in this study and approval from the Regional Committee for Medical and Health Research Ethics was not required.

## RESULTS

Of the 69 hospitals that responded in this study, 35 had performed one or more patella stabilizing procedures during 2023, Figure 1. A total of 532 patellar stabilization procedures were performed in Norway in 2023, giving an incidence rate of 9.6 per 100,000 people. The number of procedures performed by each hospital varied from 1 to 94, with the majority of hospitals (*n* = 26) performing between 5 and 20 procedures in 2023. Forty‐eight percent of the procedures were performed by 4 hospitals, including three large University hospitals and one private hospital, all of whom reported more than 40 yearly procedures.

**Figure 2:**
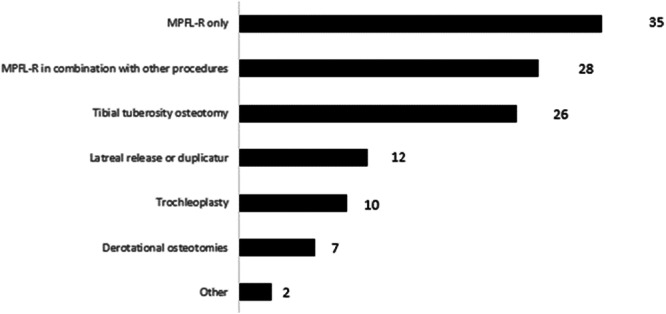
Number of hospitals that performed the different surgical procedures.

All hospitals performed MPFL reconstruction (*n* = 35), 28 hospitals included bony procedures in their approach, while seven hospitals also performed derotational osteotomies in femur (Figure 2). Furthermore, there was a large variation in the procedures performed by the hospitals. Nearly half of the responding hospitals (43%) had operated on children with open physis. Of these, three (20%) performed osseous procedures when the physis was not closed.

The preferred graft for MPFL‐R was gracilis, followed by semitendinosus. The most common method of graft fixation on femur was a interference screw followed by the adductor sling technique [32], while the preferred method of fixation on the patella was through a drilled tunnel (Table 1).

**Table 1:** Fixation methods for the reconstructed MPFL on femur and patella, *n* = 35.

	Fixation femur	Fixation patella
Interference screw	21 (60%)	2 (6%)
Adductor sling	9 (26%)	
Anchor	3 (9%)	4 (11%)
Drill tunnel		24 (69%)
Suture		4 (11%)
Other	2 (6%)	1 (3%)

*Data are presented as mean.

In 20% of the hospitals (Table 2) the use of an orthosis for up to 6 weeks after surgery was routinely recommended, and the procedure for which orthoses were most commonly used was tibial tuberosity osteotomy (86%). Restricted knee flexion after MPFL reconstruction was recommended for up to 8 weeks by 86% of the responders. All of the 26 hospitals that performed bony procedures recommended restricted weightbearing after tibial tuberosity osteotomy and 73% recommended it after other bony procedures (trochleoplasty and derotational osteotomies). Almost half of reporting hospitals (48%) recommended weightbearing restrictions for up to 6 weeks, and 48% recommended restrictions for 6 to 8 weeks (Table 2).

**Table 2:** Postoperative follow‐up recommendations.

Routines	Percent (*n*)
Recommended orthosis	20% (7)
Restricted knee flexion after MPFL	86% (30)
Restricted weightbearing after tibial tuberosity osteotomy	100% (26)
Restricted weightbearing after other bony procedures (trochleoplasty and derotational osteotomies)	73% (19)

Abbreviation: MPFL, medial patellofemoral ligament.

Follow‐up appointments ranged from no postoperative assessment (*n *= 1) to 6 to 8 weeks for most patients, with the longest follow‐up being 6 months.

## DISCUSSION

The findings of this study provide valuable insights into the incidence and treatment strategy for patellar instability in the Norwegian healthcare system. With a total of 69 (92%) hospitals responding, the data reveals a significant engagement in patellar stabilization, with 35 hospitals performing these procedures in 2023. The overall incidence rate of 9.6 per 100,000 population indicates a notable prevalence of patellar stabilizing surgery.

The substantial variation in the number of procedures performed per hospital, highlights the differences in practice patterns between hospitals. Most hospitals performed between 5 and 20 procedures. This could be attributed to several factors, including the volume of patients presenting with patellar instability in the geographic area of the different hospitals, the expertise of the surgical teams, and the availability of resources. The current investigation did not inquire the share of patients that were not offered surgery although presenting with patellar instability.

Furthermore, the fact that over half of the responding hospitals (57%) did not treat children, suggests that these patients are being referred to larger university hospitals. Another interesting finding was that there seems to be some diversity in the procedures used in the surgical approach when treating children, with most (80%) hospitals performing soft tissue procedures on patients with open physis. This aligns with the current trends, as bony procedures (osteotomy/trochleoplasty) are not commonly recommended in paediatric patients with patellar instability and open physis [12].

The current findings support the use of MPFL reconstruction as the primary surgical intervention. All responding hospitals perform this procedure, which is in line with current surgical approach trends [11, 12, 34]. The choice of graft, predominantly the gracilis tendon, is consistent with the current literature, which states that the semitendinosus, gracilis and quadriceps tendons are all acceptable options [12, 34]. Furthermore, graft diameter is not important, and the choice should be based on maximizing the use of the graft itself and whatever graft available [12, 34]. Notably, graft fixation methods varied significantly, with screws being the most common method for fixation on the femur (60%) and drill tunnel being the preferred method for fixation on the patella (69%). This reflects the current literature in this area, which shows that no fixation method for the graft to the patella or femur is superior [12, 34]. The variation in fixation techniques may reflect different institutional protocols or surgeon preferences, emphasizing the need for further investigation into the long‐term outcomes associated with each fixation method. Standardizing fixation techniques could potentially enhance surgical outcomes and reduce variability in postoperative recovery. A national registry would be ideal for providing more insight into this aspect.

Postoperative rehabilitation protocols varied considerably between hospitals. This reflects the current literature where no evidence‐based rehabilitation protocol exist after patella stabilizing surgery [5, 13, 15]. The recommendation by 85% of hospitals for restricted knee flexion for up to eight weeks aligns with the goal of protecting the reconstructed graft while allowing for adequate healing [16, 21]. Furthermore, only one out of five used orthosis also for only soft tissue procedures while most hospitals seemed to agree that orthosis should be used after TTO. The high adherence rate (86%) to orthosis use after tibial tuberosity osteotomies suggests that surgeons may be more cautious with procedures involving significant bony alterations. This could imply the need to develop a more comprehensive approach to rehabilitation that factors biological healing following bony procedures. The argument for using a brace is to promote stability and healing—and to prevent unwanted incidents that could compromise the healing after surgery [16]. Although the effect of bracing remains unclear [13], investigations into accelerated protocols (including no/minimal postoperative bracing and weight‐bearing restrictions) have shown promising results compared to more restrictive protocols [19, 22].

The variability in weightbearing restrictions postoperatively, particularly after bony procedures, indicates a lack of consensus in practice and in the literature [12, 15]. While the majority of hospitals recommend weightbearing restrictions for six to eight weeks, the absence of a standardised approach may lead to inconsistencies in patients' recovery trajectories.

The findings of this study have several implications for the clinical management of patellar instability. First, the observed variations in procedural approaches and postoperative care emphasise the need to develop evidence‐based guidelines to standardise practices across hospitals. Such guidelines could help to ensure that patients receive optimal care, regardless of the institution in which they are treated.

The current study underpins the need to establish a national patellar instability registry to reduce the variation in patient care, facilitate standardization of care with optimised treatment protocols for patellar instability, ensuring that patients receive consistent and evidence‐based care regardless of where they are treated. In addition, a national patellar instability registry can contribute to the understanding, treatment, and management of this condition, ultimately leading to better outcomes for patients affected by patellar instability.

There are some limitations to consider. First, due to the nature of survey‐based research, the list of variables is limited, which restricts the level of detail that can be collected. Second, we did not ask about the technique used when performing trochleoplasty, such as whether a thin or thick flap was used and whether the procedure was open or arthroscopic. Third, data was collected using a simple questionnaire sent out to the hospitals. No formal survey of the different hospitals' records was performed. Yet, the respondents reported that their replies were based on data extracted from the hospital's records. Fourthly, no data on patient characteristics were collected meaning that we cannot determine whether the same surgical approach is used in different hospitals for patients with similar anatomical deviations. This raises the question of whether the choice of surgical approach is based on geographical variation, patient characteristics, or both.

## CONCLUSION

This study provides a comprehensive overview of the current incidence and treatment strategy for patellar instability in Norway. The significant variability in the number of surgeries per year, choice of surgical procedures, postoperative restrictions, and follow‐up practices highlights the need for further research and the establishment of standardized guidelines. A national registry is ideal to address these discrepancies and will further provide enhance patient outcomes and ensure that individuals suffering from patellar instability receive the highest quality of care. Future studies from such a registry should aim to evaluate the long‐term outcomes associated with different surgical approaches and postoperative protocols to inform best practices in this evolving field.

## AUTHOR CONTRIBUTIONS

**Trine Hysing‐Dahl**: Conception and design of study, acquisition of data, analysis and interpretation of data and draughting of manuscript. **Håvard Visnes**: Conception and design of study, acquisition of data and draughting of manuscript. **Truls M. Straume‐Næsheim**: Conception design of study and critically revising manuscript. **Per A. S. Waaler**: Conception design of study and critically revising manuscript. **Asle Kjellsen**: Conception of study and critically revising manuscript. **Henning A. Warø**: Conception of study and critically revising manuscript. **Christian Øye**: Conception of study and critically revising manuscript. **Ann K. Hansen**: Acquisition of data and critically revising manuscript. **Alexander Tandberg**: Acquisition of data and critically revising manuscript. **Andreas Persson**: Conception design of study and critically revising manuscript. **Eivind Inderhaug**: Conception and design of study and critically revising manuscript.

## CONFLICT OF INTEREST STATEMENT

The authors declare no conflict of interest.

## ETHICS STATEMENT

The authors have nothing to report.

## Data Availability

Data are available on reasonable request. Quotations and further details are available from TH‐D at Trine.Hysing-Dahl@haraldsplass.no.

## Appendix 1 Question in the online survey

1. Which hospital do you represent?
2. Has patellar stabilizing procedures been performed at your hospital in the last 3 years?
3. How many patients are operated on annually for patellar instability at your hospital?
4. Which of the following procedures related to patellar stabilizing surgery have been performed in the last year at your hospital? (fill out as accurately as you can).
5. What is the preferred graft (most commonly used) for MPFL reconstruction?
6. What is the standard fixation method for the MPFL graft on the femur?
7. What is the standard fixation method for the MPFL graft on the patella?
8. Are patients with open growth plates operated on at your hospital?
9. If yes. Are bony procedures (osteotomy, trochleoplasty) performed on patients with open growth plates at your hospital?
10. In cases of detached osteochondral fragments, is acute refixation sometimes performed instead of removal of the fragment?
11. Do you routinely recommend the use of an orthosis after patellar stabilizing procedures?
12. After which procedures is an orthosis recommended?
13. How long is the orthosis recommended?
14. Do you recommend restricted weight‐bearing/loading/joint movement after isolated MPFL reconstruction?
15. Do you recommend restricted weight‐bearing/loading/joint movement after bony procedures (osteotomies/trochleoplasty)?
16. How long are the restrictions recommended?
17. When do you routinely have postoperative controls after patellar stabilizing procedures?
